# Clinicopathological analysis and prognostic factors of 11 patients with primary non-Hodgkin lymphoma of the small intestine in a single institute

**DOI:** 10.3892/ol.2014.2209

**Published:** 2014-06-02

**Authors:** JIA-HONG CHEN, CHING-LIANG HO, YEU-CHIN CHEN, TSU-YI CHAO, WOEI-YAU KAO

**Affiliations:** Division of Hematology/Oncology, Department of Medicine, Tri-Service General Hospital, National Defense Medical Center, Taipei 114, Taiwan, R.O.C.

**Keywords:** intestine, non-Hodgkin lymphoma, Taiwan

## Abstract

The gastrointestinal (GI) tract is the most common extranodal site of involvement in non-Hodgkin lymphoma (NHL). Primary GI NHL is frequently discussed in survival analyses. Primary intestinal NHL is significantly different from primary gastric NHL with regard to its clinical features, pathological subtype, treatment and prognosis. The small intestine is involved in lymphoma less often than the large intestine. The present study aimed to analyze the clinical and pathological characteristics of primary NHL of the small intestine and its prognostic factors. A retrospective analysis was performed on clinical data from 313 cases of NHL that occurred between 1995 and 2008 in the Tri-Service General Hospital (National Defense Medical Center, Taipei, Taiwan). Among these cases, 11 cases of primary NHL of the small intestine were identified. A Cox model was used to perform the multivariate analysis. The Kaplan-Meier method was used for the survival analysis. From the 11 patients with primary NHL of the small intestine, seven patients were male (63.6%) and four patients were female (36.3%). Furthermore, nine patients (81.8%) were diagnosed with B-cell lymphoma, of which five (45.5%) were also diagnosed with diffuse large B-cell lymphoma (DLBL). Abdominal pain and/or distention were present in six (54.5%) of the patients and jejunum involvement was also observed in six (54.5%) of the 11 patients. The mean overall survival (OS) time of the 11 patients was 27.2 months and the four-year survival rate was 36.3%. The mean OS time in the patients with jejunum involvement was shorter than in those without jejunum involvement (16.9 vs. 39.6 months), although this difference was not significant (P=0.657). Surgical treatment was performed on four of the six patients with jejunum involvement due to an acute abdomen or perforation-related peritonitis. The results of the present study indicate that DLBL is the most common subtype of primary lymphoma of the small intestine, and that the site involved in NHL may affect the potential for surgery in patients with intestinal lymphoma. Furthermore, patients with primary lymphoma of the small intestine have been found to have a poor outcome compared with those with lymphoma in other regions of the GI tract. In the present study, a similar trend was observed, however, the sizes of the subgroups of primary lymphoma of the small intestine were too small for individual analysis.

## Introduction

Non-Hodgkin lymphoma (NHL) is a diverse group of blood cancer that includes any type of lymphoma with the exception of Hodgkin’s lymphoma. The latest lymphoma classification, the 2008 WHO classification, largely abandoned the ‘Hodgkin’ versus ‘non-Hodgkin’ grouping and instead lists >80 different forms of lymphoma in four broad groups (1). The gastrointestinal (GI) tract is the most common extranodal site of involvement in NHL, accounting for 5–10% of all NHL cases. Intestinal lymphomas represent 15–20% of all GI lymphomas. The stomach is the predominant location for GI lymphomas (50%), whereas intestinal lymphomas are less frequently observed in the small bowel (20–30%) (2,3).

Previous studies have shown that even though surgical resection is necessary for local disease control and preventing bleeding and/or perforation, it is rare for the procedure to completely eliminate the lymphoma when used alone (4–8). However, a surgical resection should always be attempted for localized disease. Controversy remains with regard to the management of extensive GI lymphoma. This lymphoma is commonly diagnosed at an advanced stage, and surgical treatment is only suitable for 30–40% of such patients. As a consequence, radiotherapy and adjuvant chemotherapy are essential therapeutic approaches.

The present study provides a retrospective analysis of 11 cases of primary NHL of the small intestine, presenting the clinical and pathological characteristics and analyzing the risk factors contributing to perforation of the small bowel in this type of lymphoma.

## Patients and methods

### Patients and data collection

A retrospective analysis was performed on the clinical data from 313 cases of NHL that occurred between 1995 and 2008 in the Tri-Service General Hospital (National Defense Medical Center, Taipei, Taiwan). From the 313 patients with NHL, 11 patients were confirmed to have primary NHL of the small intestine.

The clinical data collected included: Age at diagnosis, Ann Arbor stage, Eastern Cooperative Oncology Group performance status, lactate dehydrogenase (LDH) level, extranodal site and International Prognostic Index. The initial staging involved a history and physical examination, standard blood tests for LDH and other biochemical markers, chest X-rays, bone marrow aspiration and biopsies, and computed tomography of the neck, chest, abdomen and pelvis. This study was approved by the Institutional Review Board of Tri-Service General Hospital, National Defense Medical Center (Taipei, Taiwan). Patients provided written informed consent.

### Chemotherapy

A combination of cyclophosphamide, hydroxydaunorubicin, vincristine and prednisone (CHOP) plus rituximab (R-CHOP), or CHOP was administered for three or four courses following radiotherapy for localized disease, and for 6–8 courses for advanced disease. A combination of cyclophosphamide, etoposide, prednisolone and vincristine (CEOP) or CEOP minus epirubicin was administered to patients who presented with cardiac dysfunction or were older than 70 years. CHOP chemotherapy consisted of 750 mg/m^2^ cyclophosphamide as an intravenous (i.v.) infusion on day one, 50 mg/m^2^ doxorubicin i.v. on day one, 1.4 mg/m^2^ vincristine, (maximum dose, 2 mg/body) i.v. on day one and 60 mg/m^2^ prednisone per orally (p.o.) on days 1–5. CEOP chemotherapy consisted of 750 mg/m^2^ cyclophosphamide i.v. on day one, 80 mg/m^2^ epirubicin i.v. on day one, 1.4 mg/m^2^ vincristine (maximum dose, 2 mg/body) i.v. on day one and 60 mg/m^2^ prednisone p.o. on days 1–5. The dosage and schedule of rituximab included in the R-CHOP regimen was 375 mg/m^2^ every three weeks with chemotherapy.

The second-line chemotherapy regimens were a combination of ifosfamide, carboplatin and etoposide or a combination of etoposide, methylprednisolone, cytarabine and cisplatin.

### Endpoints and statistical analysis

Complete remission and partial remission were assessed according to the Japanese 327123 International Working Group criteria (9). Stable disease was defined as less than partial remission, but not progressive disease. Progressive disease was defined as the occurrence of new lesions or a 25% increase in the sum of the products of the cross-sectional diameters of all previously detected lesions.

The primary endpoint was overall survival (OS). The final date for OS was defined as the day of mortality from any cause or the last day the patient was known to be alive. OS was assessed using the Kaplan-Meier method and compared between groups using the log-rank test (10,11). All survival analyses were performed using STATA statistical software (StataCorp LP, College Station, TX, USA). P<0.05 was considered to indicate a statistically significant difference.

## Results

### Patient characteristics

Among the 11 patients with primary NHL of the small intestine, seven of the patients were male (63.6%) and four were female (36.3%). Nine of the patients (81.8%) were diagnosed with B-cell lymphoma, of which five (45.4%) were also diagnosed with diffuse large B-cell lymphoma (DLBL) (Table I). Abdominal pain and/or distention were present in six (54.5%) of the patients and jejunum involvement was also observed in six (54.5%) of the 11 patients. The clinical characteristics of the patients with and without jejunum involvement are shown in Table II.

### Treatment

Surgical treatment was performed in four of the six patients with jejunum involvement, due to an acute abdomen or perforation-related peritonitis. The rate of surgical treatment in patients with NHL and jejunum involvement was higher than in those without jejunum involvement, although this difference was not statistically significant (66.6 vs. 40%; P=0.763).

### Outcome and survival

**Figure 1:**
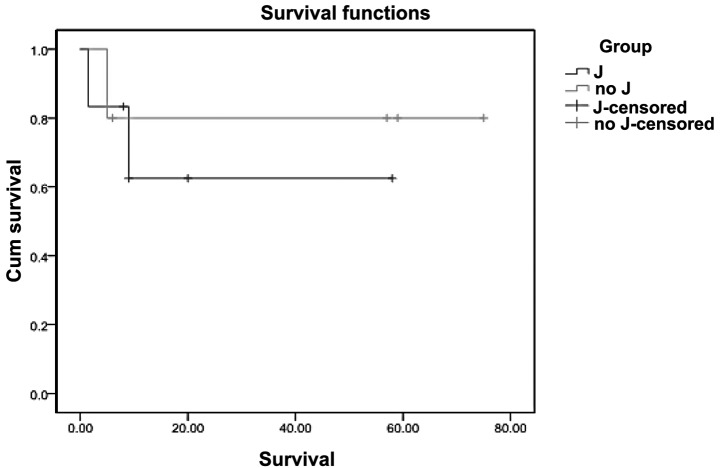
Overall survival (OS) in patients with primary non-Hodgkin’s lymphoma of the small intestine, with jejunum involvement (n=6) and without jejunum involvement (n=5). P=0.657. J, jejunum.

**Figure 2:**
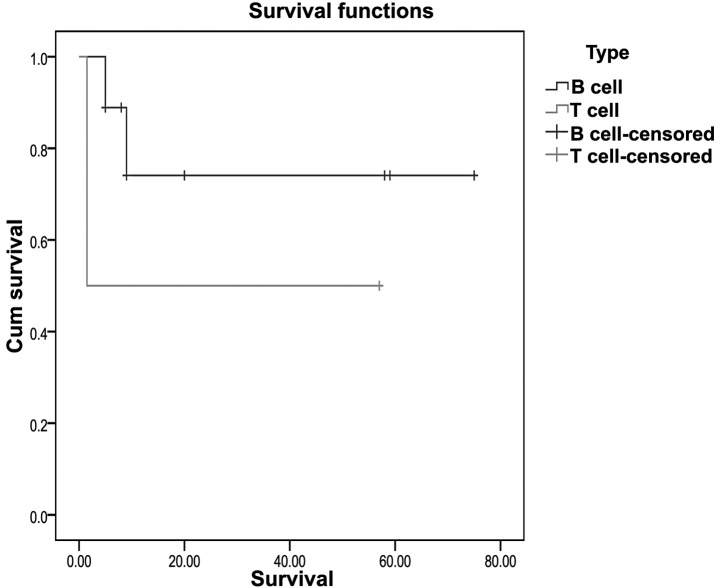
Overall survival (OS) in patients with primary lymphoma of the small intestine, with B-cell lymphoma (n=9) and T-cell lymphoma (n=2). P=0.338.

**Figure 3:**
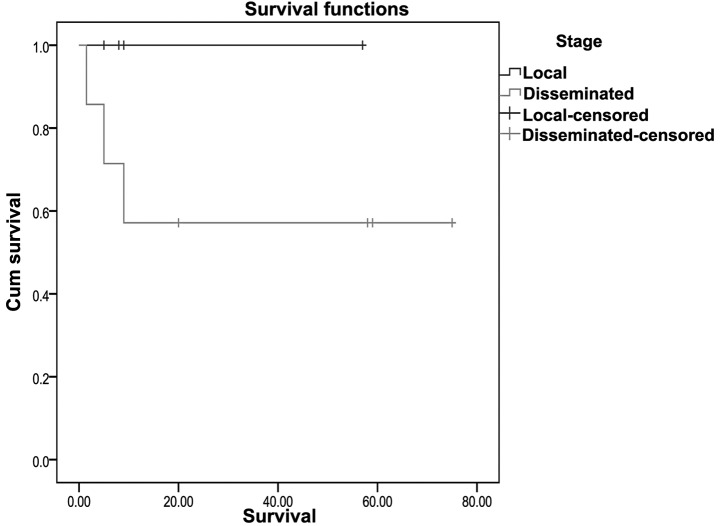
Overall survival (OS) in patients with primary lymphoma of the small intestine, with localized (n=4) and disseminated disease (n=7). P=0.202..

The mean OS time was 27.2 months and the four-year survival rate was 36.3%. The mean OS time in the patients with jejunum involvement was shorter than in those without jejunum involvement (16.9 vs. 39.6 months), although this difference was not found to be statistically significant (P=0.657; Fig. 1). Patients with T-cell lymphoma (n=2) were observed to have a poorer OS time compared with those with B-cell lymphoma (P=0.338; Fig. 2), but this difference was not statistically significant. The patients with primary lymphoma of the small intestine with disseminated disease were found to have a poorer OS time compared with those with localized disease, although this difference was also not statistically significant (P=0.202; Fig. 3).

## Discussion

The treatment strategies for intestinal NHL have yet to be established, although surgical resection is accepted as a primary treatment modality based on the results of small series of patients with intestinal NHL. Primary surgical resection has been proposed as a rational treatment choice, as it simultaneously establishes the diagnosis and reduces the tumor burden (12).

Previous studies have shown that ileocecal lymphomas frequently require surgical intervention on an emergency basis due to the anatomical site. However, even if 54% of ileocecal lymphomas require immediate surgery, 30% of non-ileocecal lymphomas also present with complications (3,13–16). Thus, lymphomas in other anatomical locations also present with complications that require emergency surgical intervention. Previous studies investigating the rate of emergency surgery in primary lymphomas of the small intestine are summarized in Table III (3,16–20). In previous studies, the rate of complications that required primary surgery was found to be between 9.3 and 23.8%, however, the rate in the present study was 54.5% among all the patients with primary NHL of the small intestine and 66.6% in the patients with NHL with jejunum involvement. In the present study, two cases had emergency surgery for intestinal perforations during chemotherapy. Thus, patients with NHL of the small intestine with jejunum involvement may benefit from primary surgical intervention followed by chemotherapy.

B-cell lymphoma is the most common type of NHL of the small intestine, and DLBL accounts for the majority of cases of B-cell NHL. Previous studies have shown that T-cell lymphomas and NHL with disseminated disease have poorer OS times, however, in the present study this difference was not statistically significant (P>0.05) (3,16–19). In the present study, the OS time was shorter in the patients with jejunum involvement compared with those without jejunum involvement.

The majority of previous studies on primary GI NHL, which has an incidence rate of one case per 100,000 individuals/year, are small, retrospective studies reporting small patient sample sizes or studies which have been performed over long periods. Such studies are often heterogeneous, combining a variety GI NHL types and using differing histological classifications, staging systems and forms of treatment.

The present study had certain significant limitations. While the study was not a randomized trial, the patient characteristics in the two groups were essentially different. Furthermore, the numbers of patients and the follow-up times were limited. A longer follow-up may stabilize the trends and allow conclusions to be made.

The results of the present indicate that DLBL is the most common subtype of NHL in primary lymphoma of the small intestine and also show that the involvement site in NHL may be an independent factor affecting surgical potential in patients with intestinal lymphoma. The outcome for patients with primary lymphoma of the small intestine is poor compared with that for patients with lymphoma in other locations in the GI tract. The present study identified that NHL with jejunum involvement may require primary surgical intervention following chemotherapy, however, the sizes of the subgroups of primary lymphoma of the small intestine were too small for individual analysis.

## Figures and Tables

**Table I:** Clinical data of 11 patients with primary NHL of the small intestine.

Case	Gender	Age, years	Involved site	Cx	Type	Stage	ECOG status	IPI	Surgery	C/T (cycles)	Outcome (months)
1	M	47	D, J, I	T	EATL	III	1	3	Y (Ru)	CHOP (1)	DOD (1.5)
2	M	75	D	B	Mantle	IV	4	5		CHOP (2)	DOD (5)
3	M	21	D	T	Anaplastic	II	1	2	Y (AP)	CHOP (6) ESHAP	AWD (57)
4	M	63	I	B	DLBL	III	1	3	Y (AP)	CHOP (6)	AWD (59)
5	F	81	J	B	Follicular	IV	2	4		R-CHOP	AWD (58)
6	M	47	J	B	DLBL	IV	2	4	Y (AP)	R-CHOP (2) R-ICE (2)+ ASCT	DOD (9)
7	F	51	D	B	DLBL	III	1	3		R-CHOP (6)	AWD (75)
8	F	80	J	B	DLBL	III	1	4		R-COP (6)	AWD (20)
9	M	87	J	B	Marginal zone	I	3	4	Y (AP)	Ex	AWD (9)
10	F	50	J	B	Follicular	II	1	2	Y (Ru)	R-CHOP R-COP	AWD (8)
11	M	17	I	B	DLBL	II	1	1		CEOP (2) R-CHOP (4)	AWD (6)

NHL, non-Hodgkin lymphoma; D, duodenum; J, jejunum; I, ileum; Cx, cell type of lymphoma; DLBL, diffuse large B cell lymphoma; EATL, enteropathy-associated T-cell lymphoma; Y, yes; AP, abdominal pain; Ru, rupture; C/T, chemotherapy; Ex, endoxan; CHOP, cyclophosphamide, doxorubicine, vincristine, prednisone; CEOP, cyclophosphamide, Epirubicin, vincristine, prednisone; COP, cyclophosphamide, vincristine, prednisone; R, Rituximab; ASCT, allogeneic stem cell transplantation; ESHAP, etoposide, methylprednisolone, cytarabine, and cisplatin; ICE, ifosfamide, carboplatin and etoposide; AWD, alive with disease; DOD, died of disease; ECOG, Eastern Cooperative Oncology Group; IPI, International Prognostic Index.

**Table II:** Patients with primary NHL of the small intestine, with or without jejunum involvement.

	Jejunum involvement	
	
Parameter	Yes (n=6)	No (n=5)
Age, years		
Mean	65.3	45.4
Range	47–87	17–75
Median	65	51
Gender, n		
Male	3	4
Female	3	1
Cell type, n		
B	5	4
T	1	1
ECOG, n		
0–1	3	4
≥2	3	1
IPI, n		
1–2	1	2
3–5	5	3
Surgery		
Yes, n	4	2
No, n	2	3
Surgical rate, %	67	40
Outcome		
OS time, months	16.9	39.6
4-year OS, n/total n (%)	1/6 (17)	3/5 (60)

NHL, non-Hodgkin lymphoma; OS, overall survival; ECOG, Eastern Cooperative Oncology Group; IPI, International Prognostic Index.

**Table III:** Comparison of the rates of emergency surgery in primary non-Hodgkin’s lymphoma of the small intestine reported in previous studies.

First author/s, year (ref.)	Small intestine	Jejunum	Complications requiring primary surgery [jejunum]	Emergency surgery rate, n/total n (%) [jejunum]
Turowski and Basson, 2004 (15)	21	NA	5	5/21 (23.8)
Koch *et al*, 2001 (2)	32	NA	3	3/32 (9.4)
Daum *et al*, 2003 (16)	83	30	19	19/83 (22.9)
Ibrahim *et al*, 2001 (17)	37	-	-	-
Kako *et al*, 2009 (18)	23	3	3	3/23 (13.0)
Kim *et al*, 2011 (19)	92	17	12	12/92 (13.0)
Present study	11	6	6	6/11 (54.5)
			[4]	[4/6 (66.7)]
